# Development of interface-dominant bulk Cu/V nanolamellar composites by cross accumulative roll bonding

**DOI:** 10.1038/srep40742

**Published:** 2017-01-17

**Authors:** L. F. Zeng, R. Gao, Z. M. Xie, S. Miao, Q. F. Fang, X. P. Wang, T. Zhang, C. S. Liu

**Affiliations:** 1 Key Laboratory of Materials Physics, Institute of Solid State Physics, Chinese Academy of Sciences, Hefei 230031, China; 2 University of Science and Technology of China, Hefei 230026, China

## Abstract

Traditional nanostructured metals are inherently comprised of a high density of high-energy interfaces that make this class of materials not stable in extreme conditions. Therefore, high performance bulk nanostructured metals containing stable interfaces are highly desirable for extreme environments applications. Here, we reported an attractive bulk Cu/V nanolamellar composite that was successfully developed by integrating interface engineering and severe plastic deformation techniques. The layered morphology and ordered Cu/V interfaces remained stable with respect to continued rolling (total strain exceeding 12). Most importantly, for layer thickness of 25 nm, this bulk Cu/V nanocomposite simultaneously achieves high strength (hardness of 3.68 GPa) and outstanding thermal stability (up to 700 °C), which are quite difficult to realize simultaneously in traditional nanostructured materials. Such extraordinary property in our Cu/V nanocomposite is achieved via an extreme rolling process that creates extremely high density of stable Cu/V heterophase interfaces and low density of unstable grain boundaries. In addition, high temperature annealing result illustrates that Rayleigh instability is the dominant mechanism driving the onset of thermal instability after exposure to 800 °C.

Future nuclear fusion reactors demand structural materials that can maintain their properties in extreme conditions, such as high temperature, high stress, and intense radiation fluxes^1^,^2^,^3^. These increased demands for future technologies cannot be met by incremental improvements to conventional materials. For example, grain refinement produced by severe plastic deformation (SPD) can greatly improve the strength^4^,^5^. However, this high strength and fine nanostructure will be lost when the material exposed to a moderately high temperature^6^,^7^,^8^. Significantly, both the extraordinary strength and the low thermal stabiity are a consequence of an unusually high density of interfaces in the SPDed metals. Therefore, materials design is not only to process bulk materials with refined nanostructure but also to develop materials containing high fraction of interfaces that are stable at extreme environment. As compared to nanocrystalline materials produced by SPD, which contain a high density of disordered and high-energy grain boundaries, nanocomposites comprised of two immiscible phases, can lead to bimetal interfaces that are sharp and potentially ordered in atomic structure^9^,^10^,^11^.

Recently, nanolayered composite films comprised of immiscible phases, simultaneously achieve high strength^12^,^13^, high thermal stability^14^,^15^ and excellent irradiation tolerance^16^,^17^. Such outstanding properties are attributed to the extremely high density of heterophase interfaces, as well as the particular atomic arrangement at the interfaces^11^,^17^,^18^,^19^. The majority of research in this area has been conducted on these nanolayered composite fabricated using a bottom-up approach such as physical vapor deposition (PVD) or other deposition methods. These synthesis techniques offer a high degree of control but are not suitable for large-scale industrial fabrication due to the low deposition rates. Therefore, realizing these outstanding material properties in structural applications requires methods of fabricating nanocomposites in the bulk form.

The severe plastic deformation method of accumulative roll bonding (ARB) has recently been developed as a fabrication method to create bulk nanolayered composites of two dissimilar metals with layer geometry similar to the PVD layer morphology^20^. The ARB process involves multiple cycles of rolling, cutting and stacking and re-rolling^21^. Advantageously, this technique is an industrially scalable processing route for creating bulk nanolamellar composites with controllable average individual layer thicknesses. Recent investigations on ARB-fabricated nanolayered sheets mainly focused on the Cu/Nb system and this ARB Cu/Nb composite, like the nanolayered composite films produced by PVD, possesses continuously layered, fine-scale micro-structures and excellent resistance to extreme conditions^10^,^22^,^23^,^24^,^25^,^26^. These exciting results encourage further studies to extend these superior structural and amazing properties to other material system, especially for the main candidate material for future nuclear fusion applications.

V-based alloy is considered as promising material candidates for future nuclear fusion structural applications due to its lower neutron cross section, and reduced radiation induced swelling^27^,^28^,^29^. Moreover, similar to Cu/Nb system, Cu/V is also an immiscible system and the heterophase interfaces in Cu/V nanolayer were indicated to be effectively reduce the concentration of radiation induced point defects^17^. Fu *et al*. reported that Cu/V interfaces can effectively reduce the overall concentration of He bubbles and swelling, and the magnitude of radiation hardening and swelling was confirmed to be significantly lower in Cu/V nanolayers as the layer thickness is reduced from 50 nm to 2.5 nm^30^,^31^,^32^. Therefore, expanding ARB technique to develop interface-dominated bulk Cu/V nanolamellar composites, allows for potential application for structural materials in future nuclear fusion reactors.

The main obstacle to fabricating bulk Cu/V nanolamellar composites with continuously nanometer-scale layers is the development of edge cracks and plastic instabilities during the repeated rolling process. In our previous work^33^, we reported that cross accumulative roll bonding (CARB) combined with an intermediate annealing process can effectively minimize plastic instabilities and the formation of edge cracks, and this technique was successfully exploited to prepare high performance bulk Cu/Ta multilayer composite sheets with a minimum individual layer thickness of 50 nm. On this basis, interface-dominant bulk Cu/V nanolamellar composite sheets were developed for the first time via CARB in this study. The evolution of the layer morphology of the CARB Cu/V composites was investigated with decreasing individual layer thickness ranging from micrometer to nano-scale. A new electron diffraction based characterization method, precession electron diffraction (PED)^34^,^35^, was employed to obtain texture distributions, grain size and shape and layer thickness statistics information in the CARB Cu/V nanocomposites. This new method not only provides data similar to that of electron backscatter diffraction (EBSD) studies, but also has finer resolution than EBSD, especially for severely strained metals. The mechanical properties and thermal stability of bulk Cu/V nanolamellar composite were evaluated and the results show that the CARB bulk Cu/V nanocomposite with layer thickness h = 25 nm exhibits both high-strength and high thermal stability. The microstructure stability of the h = 25 nm Cu/V nanocomposite was studied after exposure to different high temperatures, and the results show that Rayleigh instability is the dominant mechanism driving the onset of thermal instability.

## Results and Discussion

### Microstructural evolution of the Cu/V multilayers composite during CARB process

Using CARB technique, bulk Cu/V multilayers composites with controlled layer thickness were successfully fabricated for the first time. These multilayers composites have individual layer thicknesses h ranging from 300 μm down to 25 nm, corresponding to a total strain between 1.39 and 12.24.

**Figure 1:**
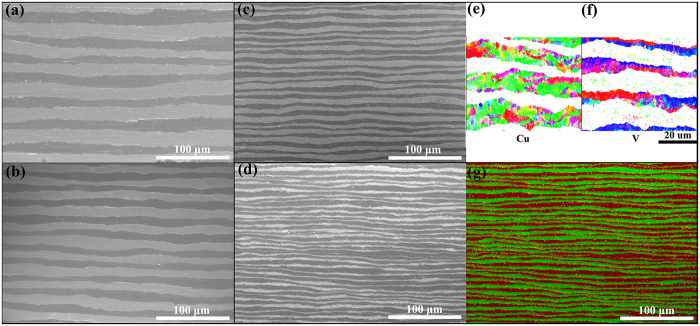
SEM cross-sectioning images of the CARB Cu/V multilayer composite with controlled individual layer thickness h: (**a**) h = 38.0 μm, (**b**) h = 19.0 μm, (**c**) h = 9.0 μm, and (**d**) h = 4.0 μm. EBSD-based inverse pole figure (IPF) maps (h = 9.0 μm) for (**d**) Cu layers and (**e**) V layers, respectively. (**g**) EDS-mapping image of layer thickness h = 4.0 μm.

For layer thickness h > 1 μm, the typical microstructure of CARB Cu/V nanocomposites are presented in Fig. 1 via SEM cross-sectioning images. Figure 1a–d show some examples of layer morphology for the CARB Cu/V nanocomposites after 3–6 rolling cycles, corresponding to an average layer thickness of 38.0, 19.0, 9.0, and 4.0 μm, respectively. SEM images showing the alternating Cu and V layered morphology, where the dark phase is V and the white phase is Cu. Average layer thickness is estimated from the total sheet thickness and number of layers. As shown in Fig. 1, the thickness of every individual Cu and V layer decreased with increasing the number of CARB cycles, and good bonding was achieved between the Cu and V layers. Despite the presence of a wavy and non-planar Cu/V interfaces with diminishing layer thickness h, a controlled and continuous layered structure has been achieved in all samples. It is reported that shear bands occur frequently in the ARB multilayered composites due to the different strength and work hardening ability between constituent layers^36^,^37^,^38^. While in our CARB Cu/V multilayer composites, shear bands were not obvious in all SEM images, indicating that CARB technique combined with intermediate annealing can effectively suppress the occurrence of shear bands in comparison with the normal ARB methods. The suppression of shear banding in the CARB Cu/V multilayers appears to be a result of the change in strain path due to cross-rolling. As reported previously^33^, at a certain total strain, in contrast to the sample prepared via the ARB process, where the strain incompatibility accumulate in a single rolling-normal direction (RD–ND) section, which significantly promoted the formation of shear bands, the CARB process could effectively lower the strain incompatibility by distributing the strain evenly in the RD and transverse direction (TD). Moreover, since strain incompatibility was induced by different thickness reduction rate due to different strengths and hardening ability of constituent layers during the rolling process, considering that the original Cu plate thickness is equal to original V plate thickness, if the thickness reduction rate of the Cu layers is equal to that of the V layers during every roll-bonding process, the theoretical average thickness of the Cu layers should be twice the thickness of the V layers after stacking in accumulative roll bonding process, meaning that strain incompatibility was suppressed. Figure 1 shows that the thickness of Cu layers is approximately twice that of V layers, indicating the Cu and V layers deform uniformly. Homogeneous deformation between the Cu and V layers facilitates further refinement. The microstructure information within the Cu and V layer for h = 9.0 μm are shown in Fig. 1e,f via EBSD grain maps, respectively. As shown in Fig. 1e, the interfaces (bonding Cu to Cu) within Cu layers have disappeared to be replaced by the recrystallized Cu gains, indicating that the interfaces are well bonded through the CARB process. There are signs of recrystallizations in Fig. 1e,f, intermediate annealing at 700 °C after every roll-bonding cycle results in full recrystallization of the Cu layer and partial recrystallization of V layers. The fully recrystallized Cu layer is one to two grains thick while the V layers with grains containing significant amounts of internal misorientation. The gains in the layers exhibit larger dimensions in the rolling direction (RD) because the grain length increases in the RD and decreases in the normal direction (ND) during the rolling process. The elemental mapping image of layer thickness h = 4 μm in Fig. 1g reveals that the Cu/V interfaces are sharp, indicating that there was neither chemical reaction nor formation of other phases between the two metals.

**Figure 2:**
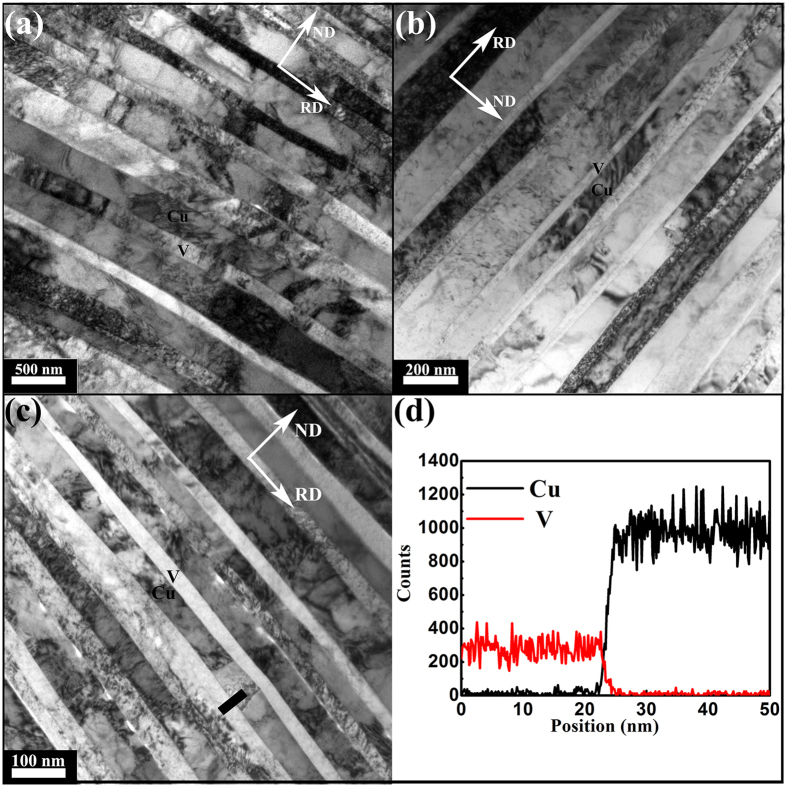
Typical bright field TEM images showing the well lamellar structure of the CARB sample was maintained for layer thickness ranging from submicrometer to nanometer length scales. Layer thickness: (**a**) h = 200 nm, (**b**) h = 100 nm and (**c**) h = 50 nm. (**d**) EDS-based line scan image of Cu/V interface for layer thickness h = 50 nm CARB material, the sharp transition between Cu and V layer exhibits chemically distinct interface.

The layer is further refined with respect to continued straining. Figure 2 presents typical TEM micrographs of CARB Cu/V nanocomposites varying individual layer thicknesses from submicrometer to nano length scale. It can be clearly seen that a continuous layer structure is preserved and the Cu/V interfaces are flat, planar, and sharp. Another notable feature of both Cu and V layers is that only one band-like grains span the layer thickness and thus all grains are connected to bimetal interfaces. Consequently, very few grain boundaries are evident in the typical TEM micrograph in Fig. 2. In all samples, the Cu layers also maintain twice thicker than V layers, indicating that the Cu and V phase deform uniformly when the layers further refined below micrometer length scales. To investigate the chemical sharpness of the Cu/V interfaces when layer thickness was refined to nano length scale, energy-dispersive spectroscopy (EDS) was utilized with the TEM. EDS line scans result in Fig. 2d shows that Cu and V phase maintain its immiscibility with no signs of chemical intermixing in spite of the extreme rolling strains during the CARB process. The TEM analysis also reveal that like the CARB Cu/Ta^33^ and ARB-CR Cu/Nb^22^ nanocomposites, deformation twins are not observed in all CARB Cu/V nanocomposites, providing evidence that CARB sample developing a different interfaces and grain orientations, which not support twinning in comparison with ARB sample^22^,^39^,^40^.

**Figure 3:**
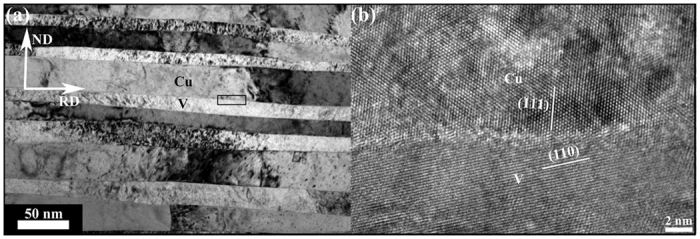
A typical layer morphology and interface structure of Cu/V nanocomposite with individual layer thickness h = 25 nm. (**a**) Typical bright field TEM image exhibiting single crystal layers with extremely high-aspect-ratio grains. (**b**) Representative HRTEM image of the predominant interface in the 25 nm CARB material, which is atomically ordered and sharp.

Figure 3a presents a typical TEM bright field image of Cu/V nanocomposites with layer thickness h = 25 nm, corresponding to a total strain of 12.23. As shown in Fig. 3a, the layers remained one grain thick with extremely high aspect-ratio gains and no ordinary grain boundaries, meaning the bimetal interface density increased and the grain boundary density decreased markedly after the extreme rolling strains caused by CARB process. This behavior is different from gain refinement caused by SPD, which leads to a high fraction of grain boundaries that are disordered in structure and high in boundary energy. Typical high-resolution transmission electron microscopy (HR-TEM) image in Fig. 3b reveals that the Cu/V interface is atomically ordered and chemically sharp. No chemical intermixing was detected at the Cu/V interface in HR-TEM image, providing further evidence that Cu/V interfaces are stable after the extreme plastic deformation. In addition, HR-TEM result also shows that the Cu and V phase achieved strong interfacial bonding by cross accumulative roll bonding process.

**Figure 4:**
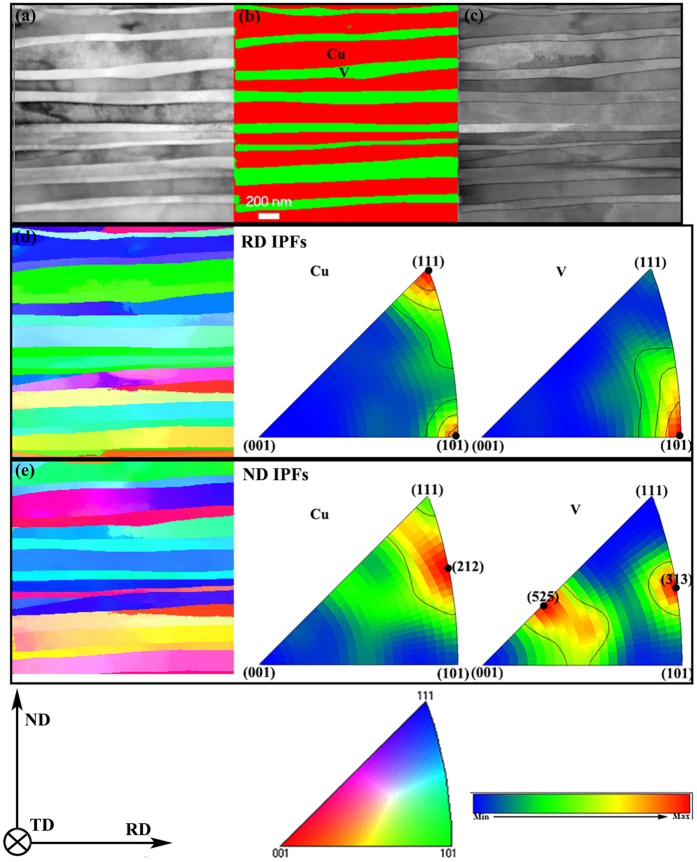
Phase mapping and orientation results of the CARB Cu/V nanocomposite with 100 nm layer thickness. (**a**) TEM bright field image for a representative PED scan region. (**b**) Phase map with Cu in red and V in green, exhibiting a fine lamellar structure with sharp Cu/V interface. (**c**) Virtual BF image from the PED system. Inverse pole figures of Cu and V, in which grains are colored with respect to their orientation as shown in the reference triangle. (**d**) RD-IPFs of Cu and V, in which the main orientation components are marked with black dots. (**e**) ND-IPFs of Cu and V, in which the main orientation components are marked with black dots. The predominant interface structures are Cu{212}<101>||V{525}<101> and Cu{212}<101>||V{313}<101>.

A relatively new characterization method, precession electron diffraction (PED), which makes use of the diffraction and scanning capabilities of thin-film samples in a transmission electron microscope (TEM), was used to study the Cu/V nanocomposites when layer thickness h = 100 nm (Fig. 4). This new method is used here to provide data similar to that of EBSD studies, but at length scales in heavily deformed metallic multilayers that are at least an order of magnitude finer than in scanning electron microscopy-based texture studies. The electron probe size was ~1 nm, and a precession angle of 0.1° was used to reduce the strong dynamical effects in electron diffraction. Data were collected over a series of five runs that encompassed areas of size 2 μm × 2 μm with a step size of 6 nm. Additional technical details of PED are available in the previous literature^34^,^35^,^41^. The phase map in Fig. 4b, where red represents Cu whereas green represents V, exhibits well laminated structure with sharp Cu/V interfaces. In the orientation maps of Fig. 4d,e, both Cu and V layers, contain elongated grains (i.e. several h long) with just one grain across the layer thickness. Rolling direction inverse pole figures (RD-IPFs) and Normal direction inverse pole figures (ND-IPFs) in Fig. 4d,e gathered from several merged PED scans to present texture information for Cu/V nanocomposite with layer thickness h = 100 nm. For V phase, the RD-IPF provides a strongest intensity in (110) orientation as shown in Fig. 4d, indicating a dominant V<110>//RD. For Cu phase, the RD-IPF in Fig. 4d shows that a strong intensity in the (111) and (110) orientation. Normal direction inverse pole figures (ND-IPFs) in Fig. 4e suggest that the (212) orientation is predominant in Cu and the (525) and (313) orientations are predominant in V. Thus, according to the PED results in Fig. 4, h = 100 nm bulk Cu/V composites contain a distribution of special interface types, Cu{212}<101>||V{525}<101> and Cu{212}<101>||V{313}<101>. These interfacial characters were different from the typical interfaces found in ARB and CARB Cu/Nb composites, where Cu{112} <111>//Nb {112} <110>^20^ and Cu {110} <112>//Nb{001}<110>^22^.

### High strength and thermal stability

**Figure 5:**
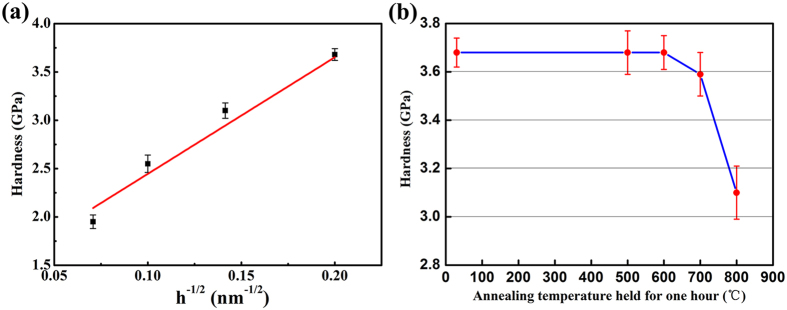
High strength and thermal stability of the bulk Cu/V nanocomposite. (**a**) Average nanohardness versus h^−1/2^, showing that strength increases with decreasing layer thickness by the Hall–Petch scaling law. (**b**) Hardness reduction as a function of annealing temperature exhibiting outstanding thermal stability of CARB Cu/V nanolamellar nanocomposite.

Mechanical strength and thermal stability of the Cu/V nanocomposites were investigated by nano-indentation hardness testing and high temperature annealing. Average hardness of CARB Cu/V nanocomposite with layer thickness from submicrometer to nanometer length scales are plotted in Fig. 5a. The hardness of Cu/V nanocomposite increases almost linearly with h^−1/2^, and reaches a peak value of 3.68 GPa at h = 25 nm. This value is twice the hardness of initial pure Cu and V. Moreover, Fig. 5a also shows that the hardness variation trend is consistent with the Hall–Petch behavior^42^,^43^ similar to that of grain boundary strengthening in nanocrystalline metals^44^. This result indicates that high densities of Cu/V heterophase interfaces in the nanocomposite can serve as effective barrier for the dislocations movement during deformation, and hence strengthen the material. Figure 5b shows the nanoindentation tests result of h = 25 nm nanocomposite before and after high temperature annealing. As revealed in Fig. 5b, the CARB Cu/V nanocomposite maintains its hardness up to 600 °C and only a slight decrement (2.6%) appears after annealing at 700 °C, corresponding to 0.64 times the melting temperature of the Cu phase. This CARB Cu/V nanocomposite exhibits outstanding thermal stability when compared to other nanocomposites prepared by severe plastic deformation. For instance, nanocrystalline Cu prepared by high-pressure torsion (HPT) undergo grain coarsening and decreases in hardness even at 50 °C because they contain a high fraction of disordered, high-energy grain boundaries^6^. This indicates that the Cu/V bimetal interfaces organized into stable low-energy interface structures during the CARB deformation process. This stable interface structure is an outcome of the immiscibility of Cu and V phase. To sum up, CARB processing is indeed an effective approach to simultaneously increase the stable Cu/V interfaces fraction and decrease unstable grain boundary fraction. Thus, CARB Cu/V nanocomposite simultaneously achieves high strength and high thermal stability.

**Figure 6:**
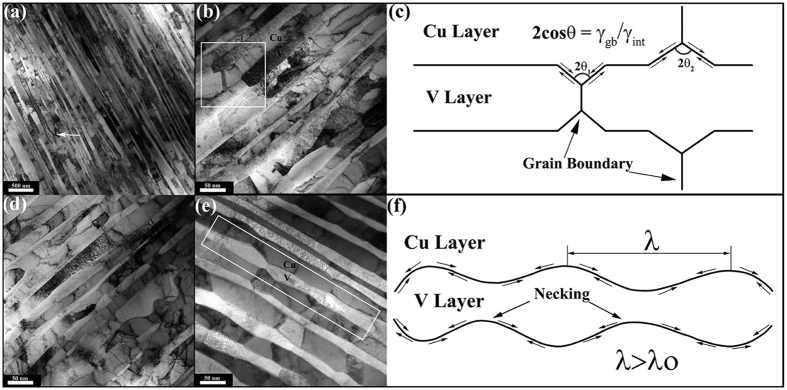
Microstructure stability of h = 25 nm Cu/V nanolamellar composites after exposure to different high temperature. (**a**) TEM micrographs showing that layer morphology after annealing at 600 °C maintains its lamellar structure with only occasional signs of pinch off. (**b**) Typical feature of layer instability due to grain boundary grooving after annealing at 700 °C for 1 h. (**c**) Schematic illustration of layer instability process driven by grain boundary grooving (the arrows indicate mass transport directions). (**d**) Typical TEM images of layer morphology after annealing at 800 °C. (**e**) Typical feature of layer instability due to Rayleigh instability after annealing at 700 °C for 1 h. (**f**) Schematic diagrams of layer instability process in CARB Cu/V composite driven by the Rayleigh instability (the arrows indicate mass transport directions).

In order to study the microstructure evolution when the Cu/V nanocomposites expose to high temperature, TEM analysis was carried out on h = 25 nm nanocomposites after annealing at 600 °C, 700 °C, 800 °C. Figure 6a presents a representative TEM micrograph of layer morphology after annealing at 600 °C for one hour. The nanocomposite maintains its lamellar structure after annealing at 600 °C, in spite of the presence of limited layer pinch-off at some localized area, as indicated by the arrow. This nearly perfect lamellar structure perfectly explains why the hardness maintained unchanged after annealing at 600 °C for 1 h. With further increasing the annealing temperature to 700 °C, obvious microstructural instability occurs with replacing the well lamellar structure. The representative features of microstructural instability are revealed in Fig. 6b,e. Figure 6b shows a typical TEM image of thermal instability driving by grain boundary grooving, which is schematically illustrated in Fig. 6c. As shown in Fig. 6b, Cu layer and V layer simultaneously develop grooves at the triple-joint junction, where the grain boundary intersects with the bimetal interfaces. The depth of the grooves is determined by the groove angle, which is related to the ratio of the grain-boundary energy γ_gb_ to interface free energy γ_int_: 2cos θ = γ_gb/_γ_int_^45^. For Cu/V nanocomposite shown in Fig. 6b, both the Cu layers and V layers developed shallow grooves with large angle θ, indicating that continued grooving to split the layers does not occur, and more stable grooves were formed^14^. This behavior is different from our previous study of CARB Cu/Ta nanolamellar composites, where deeper grooves developed in the Ta layers that lead Ta layers pinch-off^33^. The stable grooves configuration enables more excellent thermal stability of the Cu/V nanocomposite when exposure to higher temperature. Furthermore, the CARB Cu/V nanocomposite contain single crystal layers with extremely large aspect ratios and a very few grain boundaries, which further indicated that grain boundaries grooving cannot dominate in the high temperature annealed CARB Cu/V nanocomposite.

Figure 6e displays instabilities that occur within a V layer, where away from any grain boundaries and triple junctions. A similar behavior has also been recently reported for ARB Cu/Nb^25^,^46^ and CARB Cu/Ta^33^ nanolamellar multilayers, and this different thermal instability mechanism was explained by classical Rayleigh instability. As shown in Fig. 6e,f, the periodically wavy interface morphology in the V layer act as the incipient instability to driven Rayleigh instability. Incipient instability in the CARB Cu/V nanocomposite may result from the two key reasons: (i) the stress induced by the lattice mismatch and the differential thermal expansion between Cu and V during annealing and (ii) the interface fluctuations caused by the higher shear modulus of V in comparison with Cu during the CARB process. Simultaneously, a chemical potential gradient develops along the interface due to the curvature difference at the necked and flat regions, which may drive the diffusion of atoms from the necked region to the originally planar regions^25^,^46^,^47^. This process is schematically illustrated in Fig. 6f. The V layer become unstable with respect to the growth of incipient instability with wavelengths λ greater than critical wavelength λ_o_ for Rayleigh instability. As the instability progresses, the necked regions in the V layer are pinched off and the lamellar structures disappear when annealing at 800 °C, shown in Fig. 6d. In this way, the microstructure evolves to minimize its total energy.

Comparing the variation of the hardness and microstructure reveals that the CARB Cu/V nanocomposite maintain its high hardness until the ordered lamellar microstructure has degraded. Thus, the continuous Cu/V interfaces are responsible for the outstanding thermal stability by inhibiting gain coarsening when Cu/V nanocomposite exposed to high temperature.

## Conclusion

In summary, bulk Cu/V nanolamellar composites with controlled layer thickness were first successfully fabricated by cross accumulative roll bonding. Layer morphology and Cu/V interfaces are stable with respect to continued extreme straining during the CARB process. Moreover, this interface-dominant CARB Cu/V nanolamellar composites exhibit high strength and outstanding thermal stability. This outstanding property is attributed to the extremely high density of stable Cu/V interfaces and low density of unstable grain boundaries, which result from the extreme rolling strains. Such remarkable property at mechanical and thermal extremes makes this bulk Cu/V composites promising candidates for future nuclear fusion structural materials. In addition, high temperature annealing results show that the Rayleigh instability is the dominant mechanism driving the onset of thermal instability.

## Methods

### Material fabrication

Commercially available pure Cu (99.95 wt%) and V (99.99 wt%) plates were cut into dimensions of 40 mm (width) × 60 mm (length) × 1 mm (thickness). The Cu and V plates were then annealed at 600 °C for 60 min and at 900 °C for 120 min in a vacuum furnace, respectively. After annealing, a surface cleaning treatment was performed consisting of a 5-min ultrasonic acetone bath followed by wire brushing to foster bonding between the plates. Subsequently, the V plate was sandwiched between the two Cu plates and an approximate 70% reduction in layer thickness was performed on a two-high rolling mill to achieve good bonding between the Cu and V layers at room temperature (RT). After successful roll bonding, the bonded Cu/V composite was halved, cleaned and restacked. Thereafter, further roll bonding consisted of bonding Cu to Cu was performed with approximately 50% thickness reductions at room temperature. This process was repeated up to 12 times until average individual layer thickness was refined to 50 nm. Layer thicknesses finer than 50 nm were achieved via conventional rolling, that is, with no re-stacking steps and finally a sample with individual layer thicknesses of 25 nm was obtained. Different from rolling direction (RD) was kept unchanged throughout ARB process, sample was rotated 90° around the normal direction (ND) between every rolling cycle during the CARB process. The details regarding the CARB process have described in previous literature^33^. In addition, an intermediate annealing treatment was performed at 700 °C for 3 h in a vacuum furnace between every cycle before the layer structure was refined to 100 nm.

### Microstructure characterization

Layer morphology and thickness were studied by scanning electron microscope (SEM) (Sirion200, FEI, USA) equipped with an energy-dispersive X-ray spectrometer (EDS). For samples with layer thickness h < 1 μm, high-resolution transmission electron microscopy (HR-TEM) (Tecnai G2 F20, FEI) operating at 200 kV was used in order to investigate the layer morphology and interface structure. All samples were viewed along the new transverse direction (TD) and prepared using a conventional cross-sectioning method of tripod polishing, dimpling and ion milling.

Electron back scatter diffraction (EBSD) on a scanning electron microscope (SEM) was performed on cross-sectional samples to study local grain morphology and layer morphology information in the micron-scale layer thickness. Samples were prepared for EBSD by cross-sectioning, mounting, grinding, and mill polishing. Step sizes of 0.15 μm were utilized for scans on samples with h = 8 μm. A new electron diffraction based characterization method, precession electron diffraction (PED), was used to acquire microstructure and orientation information in the samples with nanoscale layer thickness where the EBSD technique is limited. Cross-sectional TEM samples were used for PED studies, with the sample viewing direction parallel to the new transverse direction (TD). Nanoscale orientation and phase mapping experiments were conducted on a FEI Tecnai F20 TEM (FEI Corporation, Hillsboro, OR) with a field emission gun and an accelerating voltage of 200 kV using the ASTARTM (NanoMEGAS, Brussels, Belgium) system. Data were collected over a series of five runs that encompassed areas of size 2 μm × 2 μm with a step size of 6 nm. Templates for Cu (a = b = c = 0.3615 nm, Fm3m) and V (a = b = c = 0.3024 nm, Im–3m) were calculated separately based on their space groups and lattice parameters. Detailed descriptions of PED process are given in previous publications^35^.

### Hardness test

All hardness tests were performed on the surfaces of the CARB-processed sheets (RD–TD plane) using a Nano-Indenter G200 mechanical testing device (Agilent) with a displacement resolution of 0.01 nm/s and a strain rate of 0.05 s^−1^. Average hardness values were calculated from 12 separate indents, which obtained for contact depths between 1000 nm and 1500 nm, corresponding to a steady-state hardness value. Moreover, the contact depths were chosen to ensure that at least two interfaces were penetrated, so that the calculated average hardness values truly represent the hardness of a composite.

### Heating treatment

In order to examine the thermal stability of the Cu/V nanolamellar composite, annealing was performed at a temperature of 500 °C, 600 °C, 700 °C and 800 °C for one hour in vacuum. The samples were cooled down also in vacuum.

## Additional Information

**How to cite this article:** Zeng, L. F. *et al*. Development of interface-dominant bulk Cu/V nanolamellar composites by cross accumulative roll bonding. *Sci. Rep.* **7**, 40742; doi: 10.1038/srep40742 (2017).

**Publisher's note:** Springer Nature remains neutral with regard to jurisdictional claims in published maps and institutional affiliations.
